# Nuclear localization of platelet-activating factor receptor controls retinal neovascularization

**DOI:** 10.1038/celldisc.2016.17

**Published:** 2016-07-12

**Authors:** Vikrant K Bhosle, José Carlos Rivera, Tianwei (Ellen) Zhou, Samy Omri, Melanie Sanchez, David Hamel, Tang Zhu, Raphael Rouget, Areej Al Rabea, Xin Hou, Isabelle Lahaie, Alfredo Ribeiro-da-Silva, Sylvain Chemtob

**Affiliations:** 1 Department of Pharmacology and Therapeutics, McGill University, Montréal, QC, Canada; 2 CHU Sainte Justine Hospital Research Centre, University of Montréal, Montréal, QC, Canada; 3 Department of Ophthalmology, Research Centre of Hôpital Maisonneuve-Rosemont, University of Montréal, Montréal, QC, Canada; 4 Department of Medicine, McGill University Health Center, Montreal, QC, Canada; 5 Department of Pharmacology, University of Montréal, Montréal, QC, Canada; 6 Experimental Surgery, Montreal General Hospital, McGill University, Montréal, QC, Canada; 7 Alan Edwards Centre for Research on Pain, McGill University, Montréal, QC, Canada; 8 Department of Anatomy and Cell Biology, McGill University, Montréal, QC, Canada; 9 Departments of Pediatrics and Ophthalmology, Faculty of Medicine, University of Montréal, Montréal, QC, Canada

**Keywords:** angiogenesis, importin, nuclear GPCR, Ptafr, rab GTPase

## Abstract

Platelet-activating factor (PAF) is a pleiotropic phospholipid with proinflammatory, procoagulant and angiogenic actions on the vasculature. We and others have reported the presence of PAF receptor (Ptafr) at intracellular sites such as the nucleus. However, mechanisms of localization and physiologic functions of intracellular Ptafr remain poorly understood. We hereby identify the importance of C-terminal motif of the receptor and uncover novel roles of Rab11a GTPase and importin-5 in nuclear translocation of Ptafr in primary human retinal microvascular endothelial cells. Nuclear localization of Ptafr is independent of exogenous PAF stimulation as well as intracellular PAF biosynthesis. Moreover, nuclear Ptafr is responsible for the upregulation of unique set of growth factors, including vascular endothelial growth factor, *in vitro* and *ex vivo*. We further corroborate the intracrine PAF signaling, resulting in angiogenesis *in vivo*, using Ptafr antagonists with distinct plasma membrane permeability. Collectively, our findings show that nuclear Ptafr translocates in an agonist-independent manner, and distinctive functions of Ptafr based on its cellular localization point to another dimension needed for pharmacologic selectivity of drugs.

## Introduction

PAF (chemical name - 1-O-alkyl-2-acetyl-*sn*-glycerol-3-phosphocholine), a proinflammatory phospholipid, was first discovered in 1960s for its role in platelet aggregation and release of histamine by the activated platelets [1]. Since then, PAF and PAF-like lipids have been shown to perform diverse functions in allergic and inflammatory processes, including airway hyper-responsiveness [2, 3], bacterial septicemia [4, 5] and anaphylaxis [6] through their actions on cardiovascular and immune systems [7]. At a cellular level, both PAF and PAF-like lipids (collectively referred to as ‘PAF’ in rest of the paper) activate a single G-protein-coupled receptor (GPCR) viz platelet-activating factor receptor (Ptafr), which is abundantly expressed in endothelial cells (ECs), platelets, neutrophils, monocytes, macrophages and various smooth muscle cells [8]. The biosynthesis of PAF in ECs is triggered by various stimuli such as thrombin, angiotensin-II, vascular endothelial growth factor (VEGF), vasopressin, and interleukin-1 (IL-1) [9]. Some newly synthesized PAF by ECs is released into the circulation but most remains intracellular, which hints towards its possible role as an intracrine signaling molecule [10]. The biological actions of PAF are short-lived (plasma half-life <15 min [6]) as it is rapidly cleared by first-pass metabolism in the liver [11] as well as by different circulating and intracellular forms of PAF acetylhydrolase [12, 13]. Nonetheless, PAF is known to produce potent vasoconstrictor, vasodilator [14, 15], hyperpermeability [16], vaso-obliterative [17] and conversely angiogenic [18, 19] effects on the vasculature.

The GPCRs, such as Ptafr, were long believed to signal by binding to their ligands exclusively at the cell surface. This notion has been challenged in recent years by discovery of functional, intracellular GPCRs in the endosomes [20], at the mitochondria [21] and at the nucleus [22, 23]. So far, more than 30 different GPCRs have been detected at the nuclei of endogenous cells and tissues [24, 25]. Our group first reported the presence of Ptafr at the nuclei of piglet brain microvascular ECs using transmission electron microscopy (TEM) and further demonstrated that the receptor is functional *in vitro* using purified EC nuclei [26]. However, the mechanism of nuclear translocation of Ptafr, like most nuclear GPCRs, remains an unsolved mystery so far. All members of GPCR family contain seven hydrophobic transmembrane domains, which necessitate the use of vesicular transport for their translocation between membranous subcellular organelles through mostly aqueous intracellular environment. After the vesicle-containing GPCR reaches its final destination, it has to fuse to the membrane of target organelle. The current evidence suggests that members of Ras superfamily of small GTPases, especially those in Rab and Arf families, are involved in the regulation of various stages of the vesicular transport as well as in the process of membrane fusion [27, 28]. Another conserved eukaryotic protein family, importin (part of karyopherin superfamily), has been recently proposed to play a role in nuclear translocation of GPCRs based on the evidence from RNA interference studies [22, 23]. Interestingly, importins recognize their cargo by the presence of a nuclear localization signal (NLS) [29] and many GPCRs, including Ptafr, contain a putative NLS [30]. Therefore, we hypothesized that nuclear translocation of Ptafr in vascular ECs is governed by specific small GTPase and importin interaction.

The GPCRs, including Ptafr, are known to signal via heterotrimeric G-protein-dependent [31] or -independent [32] pathways [26, 33]. Many components of both signaling pathways such as G-proteins [34], β-arrestin1 [35] and several GPCR kinases [36] have already been detected at the nucleus. Additionally, in the past decade, increasing studies have shown that nuclear GPCRs can perform specific functions in cultured cells [22, 23, 37, 38]. However, *in vivo* proof to substantiate these claims remains sparse. It was only recently uncovered that a GPCR, F2rl1 (F2R like trypsin receptor 1; previously known as Par2), has opposing actions depending on its localization in retinal ganglion cells [22]; in this case, nuclear F2rl1 originates from the plasma membrane (PM), whereas the origin of nuclear Ptafr is not established and does not seem to augment upon cell surface stimulation with PAF [39]. We therefore proceeded to elucidate (1) the cellular mechanisms implicated in nuclear localization of Ptafr, (2) the motifs of the receptor essential for this latter function and (3) to determine if Ptafr at different subcellular locations results in the regulation of distinct genes, which in turn translate into distinct *in vivo* vascular functions of PAF using a model of proliferative ischemic retinopathy.

## Results

### Localization of Ptafr at the nucleus is cell-type-specific

**Figure 1:**
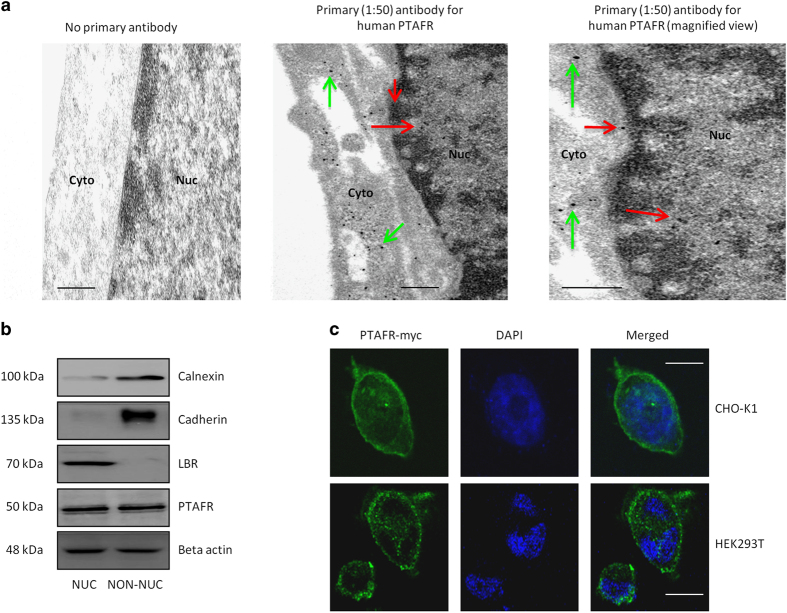
Nuclear localization of PTAFR is cell-type-specific. (**a**) TEM on cultured hRMECs. The left panel indicates no primary antibody (negative control) and secondary anti-rabbit nanogold (1:500) showing the lack of nonspecific labeling. The other two panels were treated with respective primary (1:50) and secondary (1:500) antibodies. They show specific immunogold labeling, corresponding to PTAFR in perinuclear (green arrows), nuclear envelope and intranuclear regions (red arrows). The right panel is magnified to show intranuclear localization of the receptor. The TEM images are representative of three independent experiments. Scale bar=0.5 μm. (**b**) Purity of subcellular (NUC, nuclear; NON-NUC, non-nuclear) fractions of hRMECs. The images are representative of three western blots. Organelle-specific markers used are calnexin for endoplasmic reticulum (ER), cadherin for PM and lamin-B receptor (LBR) for nuclear envelope. Beta actin is found in both the cytoplasm and nucleoplasm and was used as a loading control [111]. PTAFR is also detected in both fractions of hRMECs. (**c**) Confocal microscopy on HEK293T and CHO-K1 cells stably transfected with PTAFR-myc receptor. Nuclei are stained with DAPI (4′,6-diamidino-2-phenylindole). PTAFR shows nuclear localization in CHO-K1 cells, but not in HEK293T cells. The images are representative of three replicates. Scale bar=20 μm.

Cellular localization of Ptafr was studied in endogenous and stable-transfected cells using multiple approaches, specifically subcellular fractionation, confocal microscopy and TEM [40]. The specificities of primary anti-Ptafr and secondary nanogold antibodies were confirmed in endogenous human retinal microvascular ECs (hRMECs) as well as in transfected human embryonic kidney 293T (HEK293T) cells by comparable staining for second antibody against c-terminal myc tag-labeled PTAFR; native HEK cells lack endogenous PTAFR (Figure 1a and Supplementary Figure S1A). The purity of subcellular fractions was evaluated by immunoblotting for organelle-specific marker proteins for endoplasmic reticulum (ER), PM and the nucleus (Figure 1b) [40]. TEM revealed the presence of nuclear PTAFR in hRMECs (Figure 1a), which was confirmed on immunoreactivity of isolated nuclei by subcellular fractionation (Figure 1b), consistent with previous reports in porcine neuromicrovascular ECs [26]. Similar to HEK293T cells, Chinese hamster ovary (CHO-K1) cells showed negligible native Ptafr (Supplementary Figure S1B). However, upon stable transfection with PTAFR-myc, CHO-K1 displayed nuclear localization of the receptor, whereas HEK293T cells did not (Figure 1c and Supplementary Figure S1C). This cell-type-specific difference in the subcellular localization of PTAFR inferred possible differences in the levels of nuclear transport proteins between different cell lines.

### The nuclear translocation of Ptafr does not arise from the PM and is not agonist-dependent

**Figure 2:**
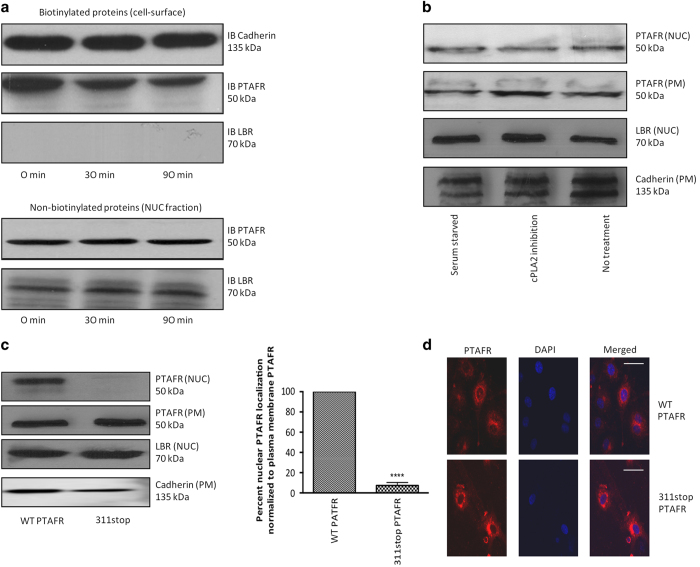
Nuclear PTAFR does not arise from PM, and requires C-terminal internalization motif. (**a**) Biotinylation of hRMECs using PM-impermeable sulfo-NHS-SS-Biotin tag. Following stimulation of with PAF C-16 for indicated time-points, biotinylated proteins were separated by binding to streptavidin beads, whereas supernatant containing non-biotinylated intracellular proteins was further subfractionated into nuclear and non-nuclear parts. IB, immunoblot. The top two panels correspond to PM cadherin and PTAFR, respectively. Nuclear protein, LBR, is absent in biotinylated fraction. The last two panels confirm the presence of LBR and PTAFR in the nuclear fraction. All western blots are representative of three independent experiments. (**b**) Effect of AACOCF3 (cPLA2 inhibitor [98]) on subcellular localization of PTAFR in hRMECs. There is no difference in localization of PM or nuclear PTAFR following AACOCF3 treatment. LBR and cadherin are used as loading controls for nuclear (NUC) and PM fractions, respectively. (**c**) Subcellular fractionation of CHO-K1 cells transfected with either wild-type (WT) or 311stop PTAFR. The quantification of three independent western blots (representative blot is shown) using NCBI’s ImageJ software (Bethesda, MD, USA) indicates ~90% reduction in nuclear signal as compared with that at PM (normalized using LBR and cadherin as loading controls for the respective fractions). ****P<0.0001. (**d**) Confocal microscopy on CHO-K1 cells transfected with WT or 311stop PTAFR. The 311stop PTAFR is present at the cell surface and perinuclear regions, but not at the nucleus. The figures are representative of three replicates. Scale bar=50 μm.

To assess whether nuclear Ptafr arises from the PM, we tagged cell surface proteins with cell-impermeable biotin (sulfo-NHS-SS-Biotin) [41]. Biotin-tagged PTAFR was not detected at the cell nucleus before and after cell stimulation with exogenous PAF C-16 [42], as the nuclear marker LBR was not detected in the biotin-tagged fraction (Figure 2a). There are two distinct sources of endogenous PAF in vascular ECs, specifically circulating (extracellular) and intracellular (retained) PAF. To remove extracellular source of PAF, we serum-starved ECs for 8 h in growth factor-devoid media. Under these conditions, stimulation of cells with PAF C-16 again did not affect cellular distribution of PTFAR (Supplementary Figure S1D). Next, to evaluate the effect of endocytosis of cell surface PTAFR on its nuclear localization, we used dynasore hydrate [43] and nystatin [44] to inhibit clathrin-dependent and -independent pathways, respectively. However, the inhibition of endocytosis had no effect on nuclear localization of PTAFR in hRMECs (Supplementary Figure S1E). The biosynthesis of PAF by remodeling pathway (major pathway during inflammation [45]) occurs in two steps. First, members of phospholipase A2 (PLA2) family generate an intermediate, lyso-PAF, from alkyl-choline phosphoglycerides. Lyso-PAF is, then, converted to PAF by the action of lyso-PAF-acetyltransferase [45]. The cytosolic phospholipase A2s (cPLA2s, also known as Group IV PLA2s) have a major role in the biosynthesis of PAF [46]. Correspondingly, pharmacologic inhibition of cPLA2 activity had no effect on nuclear localization of PTAFR in hRMECs (Figure 2b and Supplementary Figure S1F). Hence, endogenous and exogenous PAF do not affect nuclear localization of PTAFR, and nuclear PTAFR does not originate from the PM.

### The C-terminal motif of Ptafr governs its nuclear localization

Many class-A GPCRs, including Ptafr, contain putative NLS [30]. Upon bioinformatic analysis, we identified three potential internalization motifs in the C terminus of human PTAFR (Supplementary Table S1), including a ‘classical’ NLS [47]. We analyzed the function of the putative motifs by mutagenesis (^298^NNFRKH^302^, 311stop and 330stop) (Supplementary Figure S2A–C). All three mutant receptors showed similar activity compared with the wild-type PTAFR in transfected HEK293T cells, based on calcium mobilization assayed fluorometrically (Supplementary Figure S2D). The 311stop PTAFR mutant did not localize to the cell nucleus, as shown on subcellular fractionation as well as on confocal microscopy (Figure 2c and d); the other mutations did not affect nuclear localization of the PTAFR (Supplementary Figure S2E and F). Hence, the C-terminal motif (311–330 amino-acid residues in the C terminus) of PTAFR is essential for its nuclear localization.

### Perinuclear Rab11a and Ipo5 govern nuclear localization of Ptafr

**Figure 3:**
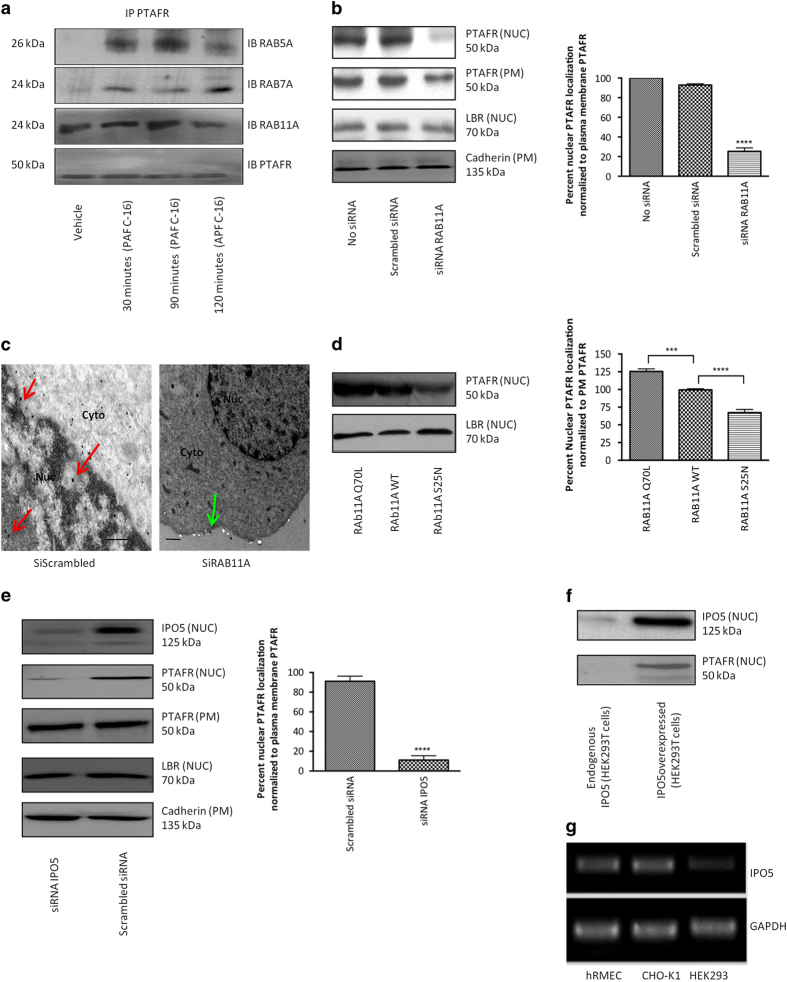
RAB11A and IPO5 govern nuclear localization of PTAFR in hRMECs. (**a**) Co-immunoprecipitation (co-IP) of PTAFR with three major rabs controlling GPCR trafficking at indicated time-points following PAF C-16 stimulation. RAB5A (top row) co-IPs with only PTAFR following stimulation (early endocytosis [112]). RAB7A (second row) also co-IPs following stimulation and peaks at around 2 h (receptor targeted for degradation [113]). RAB11A (third row) co-IPs with PTAFR at all tested time-points, even in the absence of PAF C-16 stimulation (first column). The last row shows PTAFR as a loading control. (**b**) Knockdown of RAB11A using specific siRNA in hRMECs. The quantification of three westerns using the ImageJ software reveals ~75% reduction in nuclear immunoreactivity, as compared with that at PM. PM PTAFR is slightly affected (to much lesser extent), possibly due to recycling function of RAB11A. ****P<0.0001. (**c**) TEM on hRMECs transfected with either scrambled or specific RAB11A siRNAs. RAB11A knockdown specifically affects nuclear localization of PTAFR. Red arrows indicate nuclear labeling, whereas green arrows point labeling at PM. The TEM images are representative of three replicates. Scale bar=0.5 μm. (**d**) Overexpression of constitutively active (Q70L) or dominant-negative (S25N) RAB11A mutants in hRMECs (heterogeneous expression). The Q70L and S25N mutants resulted in ~125% and ~70% nuclear localization of PTAFR, respectively, as compared with non-transfected hRMECs with endogenous RAB11A levels (set at 100%) and normalized against LBR levels in all nuclear fractions. ****P<0.0001 and ***P<0.001 (**e**) siRNA-mediated knockdown of IPO5 in hRMECs. PM PTAFR is unaffected. The quantification of western blots reveals >90% reduction in nuclear immunoreactivity, as compared with that at PM. The values were normalized against LBR and cadherin in NUC and PM fractions, respectively. ****P<0.0001. (**f**) Co-transfection of HEK293T cells with PTAFR and IPO5. The overexpression of both proteins results in nuclear localization of PTAFR in HEK293T cells. All western blots are representative of three independent experiments. (**g**) Endogenous levels of IPO5 mRNA in hRMECs, CHO-K1 and HEK293T cells. HEK293T cells show negligible endogenous expression of IPO5.

Small GTPases of the Rab family predominantly coordinate GPCR trafficking in cells [28, 48]. They are specific in cargo selection and their subcellular localization [28]. In this context, the endocytic trafficking of human Ptafr from the PM is known to be regulated by Rab5a (early endosomes) and Rab7a (late endosomes/lysosomes) [49, 50]. The members of Rab11 subfamily (Rab11a, Rab11b and Rab25) also regulate endocytic trafficking via slow recycling [51]. We co-immunoprecipitated PTAFR with aforementioned rabs (RAB5, RAB7 and RAB11A) at different time-points following stimulation of hRMECs with PAF C-16. As anticipated, RAB5A and RAB7A co-immunoprecipitated with PTAFR only after agonist stimulation, whereas RAB11A did so independent of PAF C-16 stimulation (Figure 3a). We examined if RAB11A contributed to trafficking of PTAFR to the nucleus (agonist independently). Knockdown of RAB11A in hRMECs (using small interfering RNA (siRNA); Supplementary Figure S3A) resulted in decreased nuclear localization of PTAFR by ~80% (Figure 3b) on cell fractions; this observation was confirmed on TEM (Figure 3c). Further evidence supporting a role for RAB11A in localizing PTAFR at the nucleus was revealed using constitutively active RAB11A-Q70L and inactive RAB11A-S25N mutants transiently transfected [52] in hRMECs. Nuclear PTAFR localization was increased in hRMEC overexpressing the constitutively active RAB11A-Q70L mutant, and decreased by the inactive RAB11A-S25N mutant (Figure 3d) in heterogeneous expression system.

Importins have an important role in nuclear transport of proteins [29, 53], including that of GPCRs. So far, importin β1 (Kpnb1) and transportin (Tnpo1) have been reported to be responsible for nuclear translocation of full-length GPCRs or their partial fragments [22, 23, 54]. We investigated the role of importins in nuclear localization of PTAFR. Neither PTAFR nor RAB11A co-immunoprecipitated with importin β1 in hRMECs (Supplementary Figure S3B); as for TNPO1, based on analyzed cNLS motif prediction, PTAFR lacks the PY-NLS motif required for its transport [55]. On the other hand, a lesser known member of the same family, importin-5 (Ipo5, also known as RanBP5) [56], did co-immunoprecipitate with wild-type PTAFR, but not with the 311stop mutant of PTAFR (Supplementary Figure S3C). Moreover, siRNA-induced knockdown of Ipo5 significantly reduced PTAFR expression in the nuclei of hRMECs (Figure 3e and Supplementary Figure S3D). Correspondingly, co-transfection of HEK293T cells with human wild-type PTAFR (which does not localize at the nucleus in these cells; Supplementary Figure S1C) with IPO5 did result in nuclear presence of PTAFR (Figure 3f); concordantly, PTAFR-transfected CHO-K1 cells (revealing PTAFR at the nucleus) express IPO5 (Figure 3g). Collectively, these findings indicate that localization of PTAFR to the cell nucleus requires RAB11A and IPO5.

### Nuclear Ptafr conveys functions distinct from Ptafr at the PM

**Figure 4:**
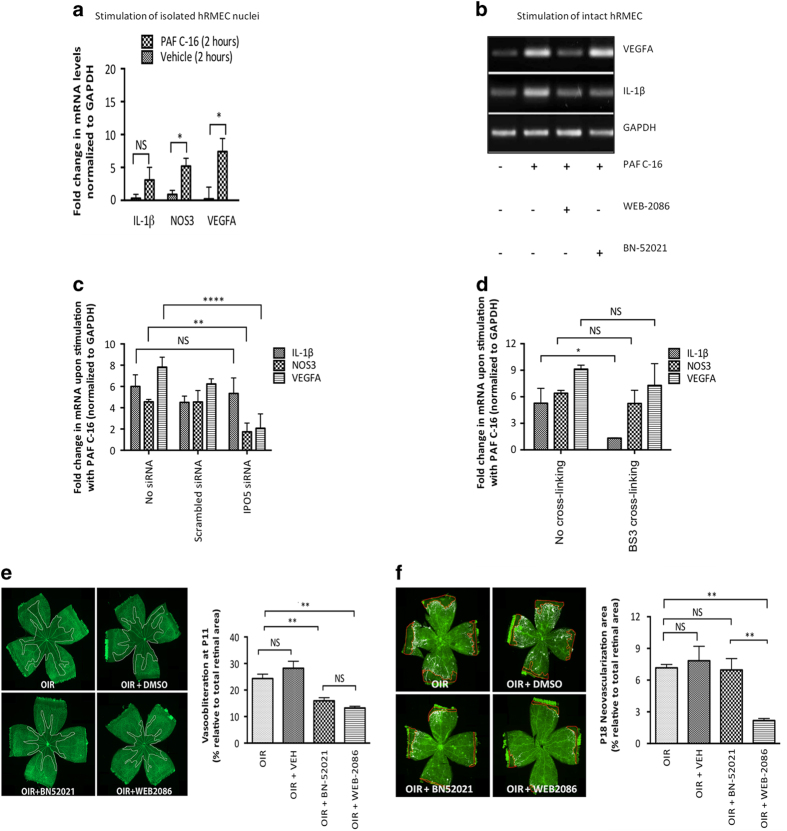
Nuclear PTAFR has functions distinct from its cell surface counterpart and the former affects retinal neovascularization in OIR. *P<0.05, **P<0.01 and ****P<0.0001. (**a**) Stimulation of freshly isolated nuclei from hRMECs with PAF C-16 (100 nm for 30 min). PAF C-16 causes significant augmentation of *NOS3* (*P*=0.015) and *VEGFA* (*P*=0.019), but not *IL1B* (*P*=0.071) levels. All values are represented as mean±s.d. The gene expression was analyzed using four independent replicates. RNA was isolated 120 min after stimulation in all conditions and gene expression was analyzed by qRT-PCR. (**b**) Stimulation of cultured hRMECs with PAF C-16 (100 nm for 30 min) with or without pre-treatment using indicated PTAFR antagonist for 30 min. The pre-treatment with membrane-permeable antagonist (100 nm WEB-2086) inhibits PAF-induced *IL1B* and *VEGFA* expression. The non-permeable antagonist (10 μm BN-52021), on the other hand, only attenuates PAF-induced *IL1B*, but not *VEGFA* mRNA levels. RNA was isolated 120 min after stimulation and analyzed by RT-PCR. (**c**) Effect of IPO5 knockdown on PAF-induced gene expression. IPO5 siRNA treatment significantly reduces PAF-induced augmentation of *NOS3* (*P*=0.004) and *VEGFA* (*P*<0.0001) levels, but has no effect on *IL1B* (*P*=0.844) levels. (**d**) Effect of chemical crosslinking on PAF-induced gene expression. The crosslinking prevented upregulation of PAF-induced *IL1B* (*P*=0.013), but had no significant difference on *NOS3* (*P*=0.997) and *VEGFA* (*P*=0.385) levels. (**e**) Retinal VO using the hyperoxia model of OIR in rats. The OIR resulted in central retinal VO at P11, as delineated by white margins in lectin-stained retinal flat-mounts (top left panel), and this was prevented by systemic administration of either Ptafr antagonists (five injections from P6 to P10) BN-52021 (*P*=0.006) and WEB-2086 (*P*=0.003) (two bottom panels), but not by the vehicle treatment (top right panel) (*P*=0.458); there was no statistically significant difference between the two antagonists (*P*>0.999). The Y axis in graph represents % of avascular retina at P11 relative to the total area (*n*=5–8 retinas per group, NS=not significant). (**f**) Retinal neovascularization using cycling model of OIR in rats. OIR-induced retinal neovascularization (top left panel) is reduced by the administration of membrane-permeable WEB-2086 (bottom right panel) (*P*=0.006), but not that of either impermeable BN-52021 (bottom left panel) (*P*>0.999) or the vehicle (top right panel) (*P*>0.999). The Y axis in graph represents % of retina with neovascularization (quantified with SWIFT-NV [110]) at P18 relative to the total area (*n*=5–8 retinas per group, NS, not significant).

Distinct functions for PTAFR depending on its general localization have been proposed, as stimulation of vascular ECs with carbamyl-PAF (synthetic PAF analog) has been shown to activate intracellular endothelial nitric oxide synthase (NOS3), but not that at the cell surface [16]. Stimulation of freshly isolated nuclei from hRMECs with PAF C-16 elicited an increase in *NOS3* and *VEGFA*, but not *IL1B* mRNA expression (Figure 4a). Consistent with these observations, pre-treatment of hRMECs with PM-impermeable PTAFR antagonist BN-52021 [57, 58] did not affect PAF-induced *VEGFA* mRNA expression, but inhibited that of the inflammatory cytokine *IL1B* (Figure 4b). Whereas membrane-permeable PTAFR antagonist WEB-2086 [59, 60], which acts on cell surface and intracellular PTAFR, prevented PAF-induced *VEGFA* and *IL1B* mRNA expression (Figure 4b), a similar profile was observed *ex vivo* on freshly isolated rat brain microvessels (Supplementary Figure S3E–G). Correspondingly, PAF-induced *NOS3* and *VEGFA* mRNA expression was abrogated upon specific knockdown of IPO5, which impairs nuclear PTAFR localization (Figure 4c), whereas *IL1B* was normally generated. Along these lines, chemical crosslinking with PM-impermeable small BS^3^ linker, which disrupts cell surface proteins [61, 62], only affected PAF-induced *IL1B* expression, but not that of *NOS3* and *VEGFA* (Figure 4d). Collectively, nuclear and cell surface Ptafr convey distinct functions, such that nuclear Ptafr stimulation induces *VEGFA* and *NOS3*, while cell surface Ptafr stimulation elicits *IL1B* mRNA expression.

### Intracellular Ptafr regulates *in vivo* angiogenesis in oxygen-induced retinopathy

Finally, we used an *in vivo* model of oxygen-induced retinopathy (OIR) in the rat [63] to show distinct functions of surface and intracellular Ptafr using BN-52021 and WEB-2086. PAF has been shown to induce both a (inflammatory thromboxane A2- and cytokine-dependent) VO [17, 64] and a (VEGF-dependent) neovascularization in the ocular tissue [65]. In line with these findings, systemic administration of either Ptafr antagonists (BN-52021 and WEB-2086) significantly reduced thromboxane A2 synthase (*Tbxas1*) and *Il1b* expression (Supplementary Figure S4C and D) and corresponding retinal VO (Figure 4e) of rats subjected to hyperoxia from postnatal days (P) 6–10; retinas were collected at P11 (Supplementary Figure S4A).

The role of intracellular PTAFR in OIR-triggered neovascularization was studied by exposing rat pups to cycling O_2_ concentrations (as described in Materials and Methods section) between P1 and P14; retinas were collected at P18 (Supplementary Figure S4B). Membrane-permeable WEB-2086, but not membrane-impermeable BN-52021 (treatment during neovascularization phase), reduced pathological neovascularization (Figure 4f) and corresponding *Vegfa* and *Nos3* expression (Supplementary Figure S4E and F). Taken together, the *in vivo* findings are consistent with *in vitro* and *ex vivo* observations on ECs and microvessels showing distinct functions for the cell surface and intracellular nuclear PTAFR, such that the cell surface receptor is mostly implicated in (inflammatory-dependent) VO, while the nuclear-comprising intracellular PTAFR is foremost involved in (VEGF-dependent) neovascularization in OIR.

## Discussion

**Figure 5:**
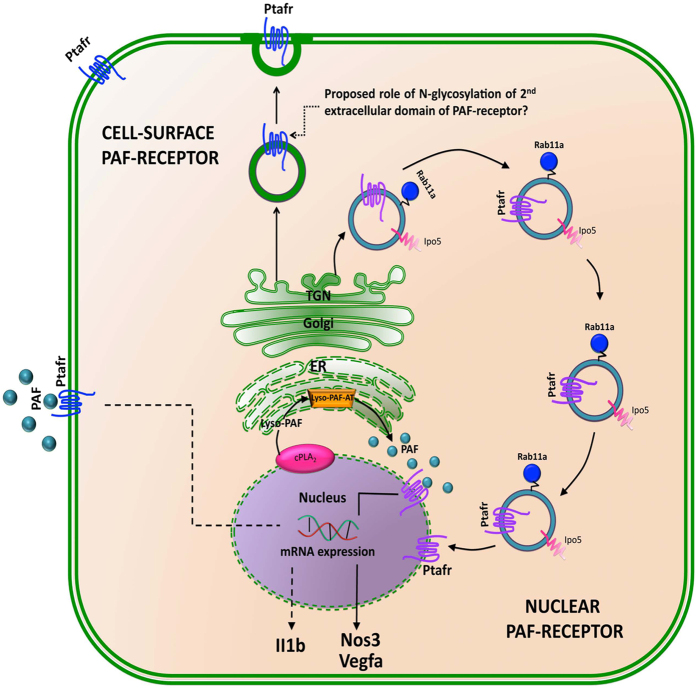
Schematic diagram showing intracellular trafficking of PTAFR. Once synthesized in ER and glycosylated in TGN, Rab11a and Ipo5 together control (pathway highlighted by blue vesicles) nuclear localization of Ptafr, possibly directly from TGN. The nuclear PTAFR can be activated by the production of local PAF from membrane phospholipids by nuclear cPLA2 [72] and ER-localized Lyso-PAF-acetyltransferase (Lyso-PAF-AT) [73]. The nuclear Ptafr, in turn, activates the expression of proangiogenic genes such as *Nos3*, *Vegfa* (indicated by solid line), whereas PM Ptafr regulated the expression of proinflammatory cytokines such as *Il1b* (indicated by dotted line). The role of N-glycosylation in the second extracellular domain of the receptor has been proposed in the cell surface targeting of Ptafr from TGN [114].

PAF-induced angiogenesis is well characterized in various experimental models of tumorogenesis [66–68]. However, cellular mechanisms governing the process in vascular ECs are poorly understood. In this study, we provide *in vitro*, *ex vivo* and *in vivo* lines of evidence for nuclear localization of Ptafr and its intracrine signaling in microvascular ECs resulting in retinal angiogenesis. We further show that Ptafr translocates to the nucleus in an agonist-independent manner via a process involving Rab11a and Ipo5. The intracellular Ptafr has functions distinct from its cognate cell surface receptor, as evidenced by impaired localization to the nucleus and effects of Ptafr antagonists with different membrane permeability, and corroborated by stimulation of isolated cell nuclei; a schematic diagram depicting our observations is presented in Figure 5. The nuclear trafficking mechanism is likely to differ among different GPCRs and needs to be investigated separately in each case.

The nuclear envelope is made up of two bi-layered phospholipid membranes (outer and inner nuclear membranes) perforated by multiprotein assemblies known as nuclear pore complexes. The outer nuclear membrane is contiguous with the ER membrane [69]. The localization of GPCRs at the nucleus has been reported for numerous receptors [25]. Yet, the mechanisms for this intracellular localization have been, for the most part, not well understood. Even though lateral diffusion is suggested to be the primary mechanism of localization for some resident nuclear membrane proteins like lamin-B receptor [70], it is unlikely to be the major pathway for translocation of GPCRs, which require post-translational (e.g., N-glycosylation) modification in the trans-Golgi network (TGN) [71]. Unlike GPCRs such as F2rl1 and Oxtr (oxytocin receptor), which translocated to the nucleus following agonist stimulation at the cell surface [22, 23], Ptafr is localized at the nucleus in an agonist- and endocytosis-independent manner, contrary to cPLA2, the key enzyme for synthesis of PAF within the cell, which does translocate to the nucleus upon activation [72], and acts on phosphatidylcholine-rich nuclear envelope [73] facilitating lyso-PAF generation. The second enzyme of the remodeling pathway, lyso-PAF-acetyltransferase, is also localized at perinuclear ER [74]. Hence, the nuclear envelope appears an ideal locus for intracrine PAF signaling. Along these lines, we previously reported that Ptafr is able to activate pertussis-toxin-sensitive heterotrimeric G proteins in purified nuclei and is responsible for gene induction of prostaglandin-endoperoxide synthase 2 (*Ptgs2*), which is a central enzyme in the biosynthesis of prostanoids under inflammatory conditions [26], particularly relevant for nuclear prostaglandin E2 signaling in microvascular ECs [75–77]. Consistent with this study, we found that PM Ptafr controls upregulation of proinflammatory vasodegenerative cytokines (*Il1b*), while intracellular Ptafr augments the expression of proangiogenic vascular factors (*Vegfa* and *Nos3*) in isolated microvessels [78, 79]. This claim is based on *Vegfa* and *Nos3* mRNA generation upon isolated nuclear stimulation, and interference with the latter in conditions whereby Ptafr does not localize to the nucleus, such as in (a) cells devoid of endogenous Ipo5 (HEK293T cells), and (b) hRMECs knockdown of Ipo5, as well as by (c) membrane-permeable Ptafr antagonist (WEB-2086).

To unravel the mechanism of translocation of Ptafr, we focused on the potential role of rab family of small GTPases. Even though members of rab11 subfamily are well-known regulators of slow endocytic recycling of cell surface proteins [80], they also perform other functions such as protein trafficking to and from TGN [81, 82], as well as during cellular processes such as phagocytosis [83] and cytokinesis [84]. Little is currently known about regulatory proteins involved in non-recycling functions of Rab11a, partly due to significant overlap between various small GTPase-mediated trafficking pathways [48, 85]. We found that both siRNA-mediated knockdown as well as transient transfection with dominant mutant form of Rab11a (S25N) significantly reduced nuclear translocation of Ptafr. Given that karyopherins act as molecular chaperones for nuclear translocation, we examined their roles in Ptafr localization at the nucleus. Out of 18 known human karyopherin-β family proteins, 11 are involved in the nuclear import (together known as importins) [29]. The most studied pathway of nucleocytoplasmic import is that a protein cargo containing a classical NLS is recognized by importin αβ heterodimer in the cytoplasm [86]. In the case of Ptafr, however, mutational disruption of the putative monopartite NLS had no effect on its nuclear localization, and well-established importin β1 did not interact with Ptafr, whereas Ipo5 did, and silencing of Ipo5 interfered with nuclear localization of Ptafr and corresponding nuclear Ptafr function (notably herein, *Vegfa* and *Nos3* induction); Ipo5 is known to be involved in nuclear import of ribosomal proteins [87] and some viral proteins [88], but there is no consensus NLS among its cargos [29]. Interestingly, we identified a new motif present at the C terminus of Ptafr (between amino acids 311 and 330), which is not known to be involved in endocytosis [89], but is essential for its nuclear localization and ensuing local Ptafr function.

To translate our *in vitro* and *ex vivo* findings revealing distinct functions for cell surface and nuclear Ptafr into *in vivo* functions, we used an ischemic retinopathy model, involving a significant contribution by PAF [17]. In the corresponding OIR model, inflammation-dependent vaso-obliteration (VO) [90] precedes an aberrant preretinal (VEGF-driven) neovascular phase. Our *in vivo* findings are consistent with cellular observations, whereby the cell surface Ptafr triggers endothelial cytotoxic *Il1b* (and *Tbxas1*) and VO, whereas the intracellular (nuclear harboring) Ptafr elicits the upregulation of *Vegfa* and corresponding aberrant preretinal neovascularization. Hence, different (and opposing) functions for Ptafr are observed, consistent with its distinct localization.

The exact orientation of nuclear GPCRs, including Ptafr, at the nuclear envelope is still unknown. It is hypothesized that G-protein-interacting C terminus would face the cytoplasm or nucleoplasm for a receptor located at the outer and inner nuclear membrane, respectively, while ligand-binding domain(s) would face the intermembrane space [25]. One could argue about access of the ligand to its activating site, but this should not be a limitation as the phospholipid ligand PAF can transverse across biomembranes and is generated intracellularly. Other than at the nuclear envelope, an abundance of Ptafr is also present within the nucleus as reported for other GPCRs [26].

In summary, our findings uncover a new mechanism for nuclear localization of a GPCR, specifically involving Rab11a (and Ipo5). We hereby demonstrate for the first time that Ptafr at the cell surface and nucleus convey distinct functions not only *in vitro* but more relevantly *in vivo*. These observations provide a new dimension to the concept of biased signaling of GPCRs, and point to the need for specific targeting of a GPCR based on its cellular localization.

## Materials and Methods

All chemicals were obtained from Sigma-Aldrich Canada Co. (Oakville, ON, Canada), unless mentioned otherwise.

PAF C-16, ginkgolide B (BN-52021) and apafant (WEB-2086) were purchased from Cayman Chemical Company (Ann Arbor, MI, USA). The (EZ-Link) Sulfo-NHS-SS-Biotin and BS^3^ reagent were purchased from Thermo Fisher Scientific Inc. (distributed by Life Technologies Inc., Burlington, ON, Canada). The Strepatavidin Sepharose was bought from GE Healthcare BioSciences (Pittsburgh, PA, USA). The RIPA buffer was purchased from Cell Signaling Technology (Danvers, MA, USA). The complete (EDTA-free) protease inhibitor cocktail tablets for lysis buffers were bought from Roche Diagnostics GmBH (Mannheim, Germany). Protein A/G PLUS-Agarose immunoprecipitation reagent was obtained from Santa Cruz Biotechnology Inc. (Dallas, TX, USA). iScript Reverse Transcription Supermix for RT-qPCR (quantitative reverse transcription PCR) and iQ SYBR Green Supermix were bought from Bio-Rad laboratories Inc. (Hercules, CA, USA).

The antibodies were purchased from the following companies (dilutions within parentheses indicate those used for the immunoprecipitation experiments, unless noted otherwise): (1) rabbit polyclonal anti-PAF receptor antibody (1:500)— Cayman Chemical Company; (2) rabbit polyclonal anti-calnexin (1:200) and monoclonal anti-lamin-B receptor (1:200) antibodies—Abcam Inc. (Cambridge, MA, USA); (3) rabbit polyclonal anti-pan-cadherin antibody (1:100)—EMD Millipore Corp (Billerica, MA, USA); (4) rabbit polyclonal anti-Ipo5 (1:100)—Acris Antibodies Inc. (San Diego, CA, USA); (5) mouse monoclonal anti-rab-11 (1:250)—BD Biosciences (San Jose, CA, USA); (6) the secondary antibodies (conjugated to horseradish peroxidase) for western blot (1:5000) were purchased from EMD Millipore Corp; (7) the secondary antibodies for immunofluroscence (1:1000) were bought from Molecular probes (distributed by Life Technologies Inc.); (8) all other antibodies (1:200) were obtained from Santa Cruz Biotechnology Inc.

The pcDNA3.1+ (Invitrogen, Burlington, ON, Canada) plasmid with cDNA clone for wild-type human PTAFR was obtained from the cDNA Resource Center (Rolla, MO, USA) (http: //www.cdna.org). The site-directed mutagenesis on PTAFR (summary is provided at the end of methods section in Supplementary Table S1) was carried out by TOP Gene Technologies Ltd (Montréal, QC, Canada) (http://www.topgenetech.com/). All mutant sequences were confirmed at the Genomics core facility, Institute for Research in Immunology and Cancer (IRIC), University of Montréal. The RAB11A constructs GFP-rab11 DN (S25N) and EGFP-Rab11A-Q70L were gifts from Richard Pagano (Addgene; plasmid no. 12678) and Marci Scidmore (Addgene, Cambridge, MA, USA; plasmid no. 49553), respectively [91, 92].

All siRNAs (Silencer Select Validated siRNAs) were purchased from Ambion Inc. (part of Thermo Fisher Scientific Inc.). The siRNA IDs were s16704 targeting human RAB11A, s7937 targeting human IPO5 and catalog no. AM4611 for scrambled siRNA (negative control). The transient transfection of siRNA (final siRNA concentration—10 nm) was carried out using Lipofectamine 3000 reagent from Invitrogen Inc. (distributed by Life Technologies Inc., Burlington, ON, Canada). The RNA or protein was harvested 72 h after transfection.

### Cell culture

HEK293T and CHO-K1 cells (American Type Culture Collection, Manassas, VA, USA) were cultured in Dulbecco's modified Eagle's medium containing 10% fetal bovine serum and 1% of penicillin (100 U ml^−1^) and streptomycin (100 μg ml^−1^). Cells reached 80% confluence before passage. Cells were transfected with appropriate plasmid DNA using linear polyethylenimine (molecular weight ~20 000) with a polyethylenimine:DNA ratio of 4:1 [ 93]. The stable selection was carried out by the addition of G418 sulfate [94] in a final concentration of 500 μg ml^−1^ for 2 weeks.

Primary hRMECs (originally from Cell Biologics Inc., Chicago, IL, USA) were a gift from Dr Pierre Hardy and were cultured in fibronectin-coated (from Roche Diagnostics, Laval, QC, USA) flasks in EC medium with EC growth supplement (ScienceCell Research Laboratories, Carlsbad, CA, USA), fetal bovine serum (final concentration: 5%) and P/S (final concentration: 100 IU ml^−1^ penicillin and 100 μg ml^−1^ streptomycin) added. The cells were used for experiments between passages 6 and 8.

### Subcellular fractionation of cultured cells

The protocol for subcellular fractionation used is identical to that we described [40]. Briefly, cells were washed with ice-cold phosphate-buffered saline (PBS) and pelleted at 500 *g* for 5 min at 4 °C (all the following steps were carried out on ice) and suspended in 2 ml lysis buffer (10 mm Trizma-HCl (pH 7.4), 10 mm NaCl, 3 mm MgCl_2_ and protease inhibitors). The suspension was homogenized with 100 gentle strokes using Dounce tissue grinder and centrifuged at 600 *g* for 10 min at 4 °C to separate nuclear fraction as a pellet, which was then resuspended in 2 ml lysis buffer containing 0.1% (v v^−1^) Nonidet P-40 alternative and protease inhibitors. Both nuclear and non-nuclear fractions were stored at −80 °C.

### Immunoprecipitation and western blotting

Total cellular proteins were extracted on ice with RIPA lysis buffer containing 20 mm Tris-HCl (pH 7.5), 150 mm NaCl, 1 mm Na_2_EDTA, 1 mm EGTA, 1% NP-40, 1% sodium deoxycholate, 2.5 mm sodium pyrophosphate, 1 mm phenylmethylsulfonyl fluoride, 1 mm β-glycerophosphate, 1 mm Na_3_VO_4_ and 1 μg ml^−1^ leupeptin. The cells were incubated on ice for 5 min and collected by scraping. Following brief sonication (3 s), supernatants were collected for use by centrifuging at 14 000 *g* for 10 min at 4 °C. The final protein concentration was determined by the Bradford method [95]. The protein samples were mixed with 4x reducing Laemmli sample buffer (250 mm Tris-HCl, 8% sodium dodecyl sulfate, 40% glycerol, 8% β-mercaptoethanol and 0.02% bromophenol blue) in 3:1 ratio (Boston BioProducts Inc., Ashland, MA, USA) [96]. The western blotting was performed using sodium dodecyl sulfate-polyacrylamide gel electrophoresis (9% acrylamide for all proteins, except rab GTPases for which 15% acrylamide was used) followed by immunoblot analysis. We analyzed western blots densitometrically using the NIH ImageJ program [97].

For immunoprecipitation, the cell lysates (500 μg) were centrifuged at 10 000 *g* for 10 min at 4 °C and the supernatant was precleared with 1 μg of appropriate control immunoglobulin G (corresponding to the host species of primary antibody used for immunoprecipitation), together with 20 μl of Protein A/G PLUS-Agarose for 1 h at 4 °C with shaking. The beads were pelleted by centrifuging at 1000 *g* for 5 min at 4 °C and incubated with 2 μg of primary antibody and 20 μl of protein A/G-agarose overnight at 4 °C on a shaker. The next day, samples were centrifuged at 1000 *g* for 5 min, washed four times with lysis buffer and finally the pellet was resuspended in 40 μl of 1x electrophoresis sample buffer (Bio-Rad Laboratories Inc.).

### Confocal microscopy

Cells were cultured in 6-well plates containing coverslips (VWR International, Ville Mont-Royal, QC, Canada). They were fixed and permeabilized with paraformaldehyde 4%, taxol (5 μm) and Triton X-100 (0.2%) for 30 min. Blocking solution (5% bovine serum albumin in PBS) was added for 1 h and was also used to dilute appropriate primary and secondary antibodies. The concentration of anti-PATFR antibody used was 1:200. Nuclei labeling was carried out with DAPI (1:3000 for 10 min) in permeabilized cells. Confocal microscopy imaging was performed on LSM 510 meta confocal microscope (Zeiss, Toronto, ON, Canada) at Sainte Justine Hospital Research Centre (Montréal, QC, Canada).

### TEM on cultured cells

The protocol for TEM is that we previously reported in detail [40]. Briefly, cells, grown on Lab-Tek chamber slides (Fisher Scientific, Ottawa, ON, Canada), were washed two times with PBS and fixed at room temperature in a fixation buffer (4% paraformaldehyde, 0.25% glutaraldehyde, 50 mm sucrose, 0.4 mm CaCl_2_ in 0.1 m sodium phosphate buffer (pH 7.4) for 30 min). Samples were washed with 1% bovine serum albumin in PBS and treated with 1% sodium borohydride in PBS for another 30 min. After carefully removing the bubbles by 4–5 washes with PBS, they were blocked with 1% bovine serum albumin and incubated with primary (1:50) and secondary (nanogold, 1:500) antibodies overnight each and washed with PBS. The second fixation was carried out with 1% glutaraldehyde in PBS for 10 min and treated with silver intensification kit reagents (Nanoprobes HQ SILVER Kit; Nanoprobes Inc., Yaphank, NY, USA). The samples were dehydrated, embedded using Epon and curated in an oven. The embedded cells were removed by peeling off the slide and then breaking off the sides of the chamber. The ultrathin sections were obtained using an ultramicrotome equipped with a diamond knife, collected on single-slot Formvar-coated grids, contrast stained with 4% uranyl acetate in water and Reynold’s lead citrate. The electron micrographs were obtained at the electron microscopy facility in the Department of Pharmacology and Therapeutics at the McGill University (Montréal, QC, Canada).

### Cell surface biotinylation

Before biotinylation, hRMECs were stimulated with PAF C-16 as described below for indicated time-points. The biotinylation protocol was slightly modified from the original version [41]. In brief, cells were washed with ice-cold PBS/CM (1x PBS, 0.9 mm CaCl_2_, 0.33 mm MgCl_2_) and incubated with 0.5 mg ml^−1^ Sulfo-NHS-SS-Biotin in PBS/CM for 30 min at 4 °C. After quenching excess (unbound) biotin with 50 mm NH_4_Cl_2_ in PBS/CM for 15 min at 4 °C, cells were lysed and biotinylated proteins (i.e., those present at the cell surface at the time of biotin treatment) were precipitated using strepatavidin beads. The non-biotinylated proteins were collected in the supernatant and were further subfractionated into nuclear and non-nuclear parts, as described previously [40].

### cPLA2 activity assay

The cPLA2 Activity Kit was purchased from Cayman Chemical Company. Following AACOCF_3_ [98] treatment at the indicated concentrations for 30 min, cells were collected and homogenized. The supernatant was collected for the assay by centrifuging at 10 000 *g* for 15 min at 4 °C. The assay [99] and analysis was performed exactly as per the manufacturer’s instructions [100]. After the assay, the absorbance was read at 405 nm.

### Fluro-4 NW calcium assay

Fluo-4 NW (No-Wash) Calcium Assay Kit was purchased from Molecular Probes. The assay is used to study GPCR function by measuring intracellular rise in Ca^2+^ levels [101] (typically mediated by GPCR coupling to heterotrimeric Gq proteins, although not exclusively [102]).

One day before the experiment, ~40 000 cells per 100 μl per well were seeded in a 96-well microplate. The following day, the dye loading solution was prepared by adding 10 ml of assay buffer and 100 μl probenecid stock solution to one bottle of Fluo-4 NW dye mix (component-A). The final concentration of probenecid was 2.5 mm.

Briefly, the medium was removed and replaced with 100 μl of the dye loading solution in each well of the plate. The plate was incubated at 37 °C for 30 min and at room temperature for additional 30 min. The cells were stimulated with 1 μm PAF C-16 (i.e., 10x normal concentration) in the Assay buffer (provided in the kit, final volume of 10 μl per well) for 30 min at room temperature. The fluorescence was measured by microplate reading fluorescence reader (EnVision Multilabel Reader; Perkin-Elmer, Waltham, MA, USA) for excitation at 494 nm and emission at 516 nm.

### Isolation of intact nuclei from cultured cells

The protocol is described in Gobeil *et al*. [77]. hRMECs were washed two times with ice-cold PBS, scraped and pelleted at 500 *g* for 5 min at 4 °C. Pellets were resuspended in lysis buffer (10 mm Tris-HCl, 10 mm NaCl, 3 mm MgCl_2_ and complete protease inhibitor added, final pH 7.4). The resulting mixture was homogenized in a Dounce tissue grinder (tight pestle; Bellco Glass Inc., Vineland, NJ, USA) with 100 strokes on ice. The homogenate was centrifuged at 700 *g* for 10 min at 4 °C. The pellet contained the nuclei and was collected in 7.5 ml lysis buffer containing 0.1% alternative to NP-40 and was left on ice for 5 min. The mixture was centrifuged at 800 *g* for 10 min at 4 °C and was washed two times with lysis buffer with NP-40 alternative. After the final wash, functional nuclei were resuspended in an incubation buffer containing 25 mm HEPES, pH 7.2, 125 mm KCl, 4 mm MgCl_2_, 2 mm K_2_HPO_4_, 400 nm CaCl_2_ and 0.5 mm ATP.

### Stimulation of cells, nuclei, microvessels with PAF C-16 and endocytosis assay

All stock solutions were prepared in dimethyl sulfoxide and stored at −20 °C and the final dilutions were freshly prepared in basal medium just before their use.

All biological samples (cells, tissues and microvessels) were starved in serum and growth factor-free endothelial cell basal medium for 8 h before the agonist stimulation. The microvessels were used for experiments within 12 h of isolation. The agonist stimulation was carried out in a basal medium with added PAF C-16 (concentration 100 nm) for 30 min. The RNA and/or proteins wee isolated at the indicated time-points. In experiments involving pre-treatment with Ptafr, antagonists, BN-52021 and WEB-2086, were used in concentrations of 10 μm and 100 nm for 30 min before the PAF C-16 stimulation, respectively [103–105]. The endocytosis inhibitors, Dynasore hydrate (80 μm) [43] and nystatin (50 μm) [44] in 1% dimethyl sulfoxide, were used for pre-treatment of hRMECs for 30 min before PAF C-16 stimulation, where indicated.

### Chemical crosslinking with BS^3^

The protocol was modified from Mattson *et al*. [61] and Angers *et al*. [62]. For chemical crosslinking, before stimulation with PAF C-16, hRMECs were treated with 1 mm BS^3^ in the reaction buffer (20 mm sodium phosphate, 150 mm NaCl, pH 8.0) for 30 min at room temperature. The reaction was stopped by treatment with 1 m Tris (pH 7.5) for 15 min, followed by washing with the basal medium two times.

### RNA isolation and qRT-PCR analysis

We extracted mRNA with Trizol (Invitrogen), synthesized cDNA and amplified it using *Taq* DNA polymerase (Invitrogen, Molecular Probes). All primers were synthesized by Alpha DNA (Montréal, QC, Canada). All new primers were designed using NCBI’s primer-BLAST program [106]. The primer sequences are provided in Supplementary Tables S2 and S3.

Before RNA isolation, retina samples in Trizol were homogenized by using Precellys24 tissue homogenizer (two runs of 5000 r.p.m. for 60 s each with gap of 90 s between the cycles). Then, samples were incubated at room temperature for 5 min. One hundred microliters of CHCl_3_ was added to each tube and was mixed for 15 s by shaking and incubated for 3 min at room temperature. Then, they were centrifuged at 12 000 r.p.m. 15 min at 4 °C and the clear liquid phase was transferred to a new tube. Two hundred and fifty microliters of isopropanol was added to each tube and incubated for 10 min at room temperature. Later, they were centrifuged at 12 000 r.p.m. for 10 min at 4 °C, followed by washing two times with cold 70% ethanol and centrifuged for 5 min at 8000 r.p.m. at 4 °C. Last, the pellet was air dried for 15 min and resuspended in 20 μl of nuclease-free sterile water. The nucleic acid quantification was carried out using NanoDrop 2000 spectrophotometer from Thermo Fisher Scientific.

The iScript Reverse Transcription Supermix for RT-qPCR (Bio-Rad Laboratories Inc.) was used for RT of RNA into cDNA (20 μl per reaction) with the cycle setting of 5 min at 25 °C (priming), 30 min at 42 °C (RT) and 5 min at 85 °C (stopping the reaction). The DNA concentration was measured using NanoDrop 2000 (Wilmington, DE, USA) and samples were stored at −20 °C.

For comparative qPCR, 2× iQ SYBR Green Supermix (Bio-Rad Laboratories Inc.) was used. Total volume of each reaction was 25 μl (12.5 μl 2× Supermix, 100 nm each of forward and reverse primers, 500 ng of cDNA and distilled water). All PCR reactions were run in triplicate. The qPCR was carried out using Applied Biosystems 7500 Real-Time PCR (Foster City, CA, USA). The thermal cycle was 50 °C for 2 min and 95 °C for 2 min (holding stage), followed by 35 cycles each of 95 °C for 15 s, 58 °C for 1 min and 72 °C for 15 s (cycling stage). The analysis was performed by the ∆∆Ct method. We normalized cDNA concentrations against GAPDH (glyceraldehyde 3-phosphate dehydrogenase) levels in each sample.

### Animal care (for *ex vivo* and *in vivo* protocols)

All experimental procedures were approved by the Animal Care Committee of the Hôpital Sainte Justine (Montréal, QC, Canada) in accordance with guidelines established by the Canadian Council on Animal Care.

### Isolation of intact microvessels from rat brain

The protocol is a modified version of the one described in Peri *et al*. [107]. Briefly, after killing the animal (4-week-old rats), the brain was perfused with PBS to eliminate blood elements. The brain was removed and immediately kept in ice-cold Krebs buffer (120 mm NaCl, 4.5 mm KCl, 2.5 mm CaCl_2_, 1 mm MgCl_2_, 27 mm NaHCO_3_, 1 mm KH_2_PO_4_, 0.01 mm sodium edetate and 10 mm glucose) and was cut into fine (<5 mm) pieces. The cut tissue was spun at 1 000 r.p.m. for 10 min at 4 °C and then homogenized gently (5–6 strokes) in ice-cold PBS containing 20% Ficoll-400 using a glass homogenizer with tight fitting glass pestle. The homogenate was centrifuged at 12 000 r.p.m. for 15 min at 4 °C. The pellet containing the microvessels was washed three or four times with 25 ml of ice-cold PBS to eliminate Ficoll and centrifuged at 2 000 r.p.m. for 20 min at 4 °C after each wash. Last, the microvessels were filtered through 70 and then 40 μm filters (Millipore, Billerica, MA, USA) to remove bigger vessels. The final filtrate was transferred to 6-well plates containing the endothelial growth medium. The microvessel preparations were assessed for purity by light microscopy and γ-glutamyl transpeptidase activity as described in Li *et al*. [108]. The fresh microvessels were used for PAF stimulation within 12 h of isolation. The RNA isolation from microvessels was carried out by the method, as described previously.

### *In vivo* OIR models

#### Retinal VO model.

To investigate PAF-induced VO, we used a well-established model of human retinopathy of prematurity—the VO model in rats, characterized by exposing the pups to 80% O_2_ during the first week of life, when vasculature is still underdeveloped and susceptible to damage caused by various inflammatory mediators, including PAF, leading to central loss of retinal microvasculature [17, 90], a hallmark of the first phase of ischemic retinopathies in humans [109]. Briefly, Sprague–Dawley albino rat pups (Charles River, St Constant, QC, Canada) were placed with their mothers in an 80% oxygen environment from P5 to P10, when normal retinal vasculature reaches the periphery. The ability of BN-52021 or WEB-2086 to curb VO was tested in four different VO groups (*n*=8 animals per group) treated from P5 to P10 as follows: VO group 1, did not receive any treatment; VO groups 2–4, were randomly selected to receive daily intraperitoneal vehicle (dimethyl sulfoxide), or 10 mg kg^−1^ per day of BN-52021 or WEB-2086, respectively, in a final volume of 50 μl. Control animals (group 5) were maintained in room air (21% O_2_) throughout the 5 days. The pups were killed on P10. Eyes were enucleated and retinas were dissected. Fifty percent of the retinas were used for RT-qPCR. In the other half, VO was evaluated in retinal flat-mounts stained with lectins by using the Image J software (Universal Imaging, Sunnyvale, CA, USA). Vaso-obliterated areas were assessed as the retinal area devoid of vasculature over the total retina. All other conditions (e.g., light exposure, temperature, feeding, and so on) were similar for all the treatment groups.

#### Retinal neovascularization model.

Within 4 h after birth, litters of Sprague–Dawley albino rats were placed with their mothers in an oxygen-regulated environment (OxyCycler A820CV; BioSpherix Ltd, Redfield, NY, USA) adjusted to alternate between 50 and 10% of oxygen every 24 h for 14 days. At P14, pups were transferred to room air (21% O_2_) for 4 days. The cycling O_2_ conditions produce the peripheral retinal neovascularization, which closely resembles the second phase of retinopathy of prematurity in humans [63]. The ability of BN-52021 or WEB-2086 to curb VO and a subsequent decrease in pathological neovascularization was tested in four different OIR groups (*n*=8 animals per group) treated from P14 to P18 (during neovascularization) as follows: OIR group 1, did not receive any treatment; OIR groups 2, 3 and 4, were randomly selected to receive daily intraperitoneal vehicle (dimethyl sulfoxide), or 10 mg kg^−1^ per day of BN-52021 or WEB-2086, respectively, in a final volume of 50 μl. Control animals (group 5) were maintained in room air (21% O_2_) throughout the 18 days. On day P18, rats were anesthetized with isoflurane (2%) and killed by decapitation. Eyes were enucleated and retinas were dissected. Neovascularization was evaluated in retinal flat-mounts stained with lectins. Fifty percent of the retinas were used for RT-qPCR. All other conditions (e.g., light exposure, temperature, feeding and so on) were similar for all treatment groups.

### Retinal flat-mounts

In all cases, the eyes were enucleated and fixed in 4% paraformaldehyde for 1 h at room temperature and then stored in PBS at 4 °C until used. The cornea and lens were removed and the retina was gently separated from the underlying choroid and sclera under a dissecting microscope. Then, the retinas were stained overnight at 4 °C with fluorescein-labeled GSL 1, isolectin B4 (Vector Labs, Burlingame, CA, USA; 1:100). Lectin-stained retinas were whole-mounted onto Superfrost/Plus microscope slides (Fisher Scientific) with the photoreceptor side down and imbedded in Fluoro-gel (Electron Microscopy Sciences, Hatfield, PA, USA) and imaged at x10 using a Zeiss (Carl Zeiss Ltd., Toronto, ON, Canada) AxioObserver.Z1. Images were merged into a single file using the MosiaX option in the AxioVision 4.6.5 software (Carl Zeiss Ltd.). Quantification of neovascularization was assessed using the SWIFT-NV method [110], which consists of a set of macros that was developed to quantify all the pixels represented by neovascular tufts and clusters, but not normal vessels in lectin-stained retinal whole mounts.

### Statistical analyses

All data are presented as means±s.d. We made comparisons between groups by one-way analysis of variance, followed by *post hoc* Bonferroni’s test for comparison between groups. *P*<0.05 was considered statistically significant.
