# Nuclear localization of platelet-activating factor receptor controls retinal neovascularization

**DOI:** 10.1038/celldisc.2016.34

**Published:** 2016-09-20

**Authors:** Vikrant K Bhosle, José Carlos Rivera, Tianwei (Ellen) Zhou, Samy Omri, Melanie Sanchez, David Hamel, Tang Zhu, Raphael Rouget, Areej Al Rabea, Xin Hou, Isabelle Lahaie, Alfredo Ribeiro-da-Silva, Sylvain Chemtob

**Affiliations:** 1 Department of Pharmacology and Therapeutics, McGill University, Montréal, QC, Canada; 2 CHU Sainte Justine Hospital Research Centre, University of Montréal, Montréal, QC, Canada; 3 Department of Ophthalmology, Research Centre of Hôpital Maisonneuve-Rosemont, University of Montréal, Montréal, QC, Canada; 4 Department of Medicine, McGill University Health Center, Montreal, QC, Canada; 5 Department of Pharmacology, University of Montréal, Montréal, QC, Canada; 6 Experimental Surgery, Montreal General Hospital, McGill University, Montréal, QC, Canada; 7 Alan Edwards Centre for Research on Pain, McGill University, Montréal, QC, Canada; 8 Department of Anatomy and Cell Biology, McGill University, Montréal, QC, Canada; 9 Departments of Pediatrics and Ophthalmology, Faculty of Medicine, University of Montréal, Montréal, QC, Canada

**Correction to:** *Cell Discovery* (2016) **2**, 16017; doi:10.1038/celldisc.2016.17; published online 12 July 2016

In the initial publication of this article, the abbreviation of the first author’s name in citation ‘K Bhosle V’ was incorrect, which should be ‘Bhosle VK’. The error has now been rectified. The article with the corrected author name in citation is now online, together with this corrigendum. Please use the citation data in the corrigendum if you cite this article.

