# Supplementary material for: Nuclear localization of platelet-activating factor receptor controls retinal neovascularization
Source: Cell Discov. 2016 Jul 12;2:16017–. doi: 10.1038/celldisc.2016.17 (PMC4941644; doi:10.1038/celldisc.2016.17)
Supplement: Supplementary Figure S1 [file celldisc201617-s1.pdf]

Supplemental figure-1

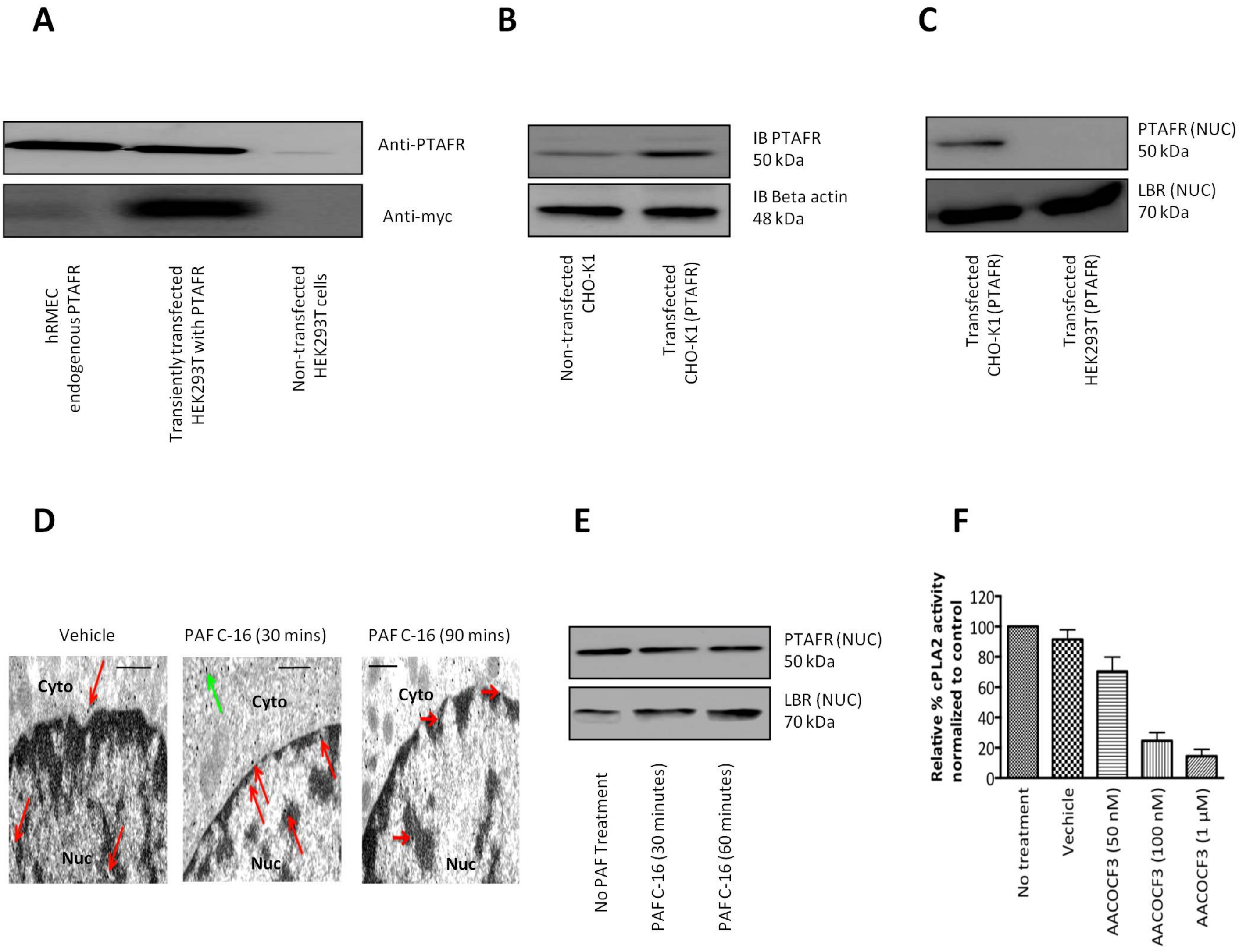

Supplemental figure-1. (**A**) The specificity of primary PTAFR antibody against its N-terminus (endogenous) or C-terminus (for myc tagged receptor). The N-terminus antibody (top panel) detects both native (in hRMEC) and transfected PTAFR (HEK293T cells). The non-transfected HEK293T cells don't express the receptor. (**B**) The expression of Ptafr in CHO-K1 cells. Non-transfected CHO-K1 cells express very little Ptafr. Beta actin was used as loading control. (**C**) Subcellular fractionation of CHO-K1 and HEK-293T cells stably transfected with PTAFR-myc. PTAFR is only found in nuclear fraction in the transfected CHO-K1 cells but not in transfected HEK293T cells. (**D**) TEM on cultured hRMEC following agonist stimulation with PAF C-16 at the indicated time-points. All tested time-points show nuclear localization of PTAFR (red arrows), while perinuclear nanogold labeling gradually increases following PAF C-16 stimulation (receptor present in recycling endosomes). TEM images are indicative of three replicates. Scale bar = 0.5 µm. (**E**) Effect of endocytosis inhibitors on nuclear localization of PTAFR. hRMEC were pre-treated with 80 µM Dynasore hydrate and 50 µM nystatin for 30 minutes to inhibit clathrin-dependent and clathrin-independent endocytosis respectively, followed by stimulation with 100 nM PAF C-16 for indicated times. The inhibition of endocytosis with or without PAF C-16 stimulation had no effect on nuclear localization of PTAFR in hRMEC. (**F**) Effect of AACOCF3 on cPLA2 activity in hRMEC. The treatment with 100 nM AACOCF3 for 30 minutes resulted in more than 80% reduction of cPLA2 activity and was used for the experiment in Figure- 2B. All western blots are representative of three independent experiments.
