# Supplementary material for: Nuclear localization of platelet-activating factor receptor controls retinal neovascularization
Source: Cell Discov. 2016 Jul 12;2:16017–. doi: 10.1038/celldisc.2016.17 (PMC4941644; doi:10.1038/celldisc.2016.17)
Supplement: Supplementary Figure S2 [file celldisc201617-s2.pdf]

Supplemental figure-2

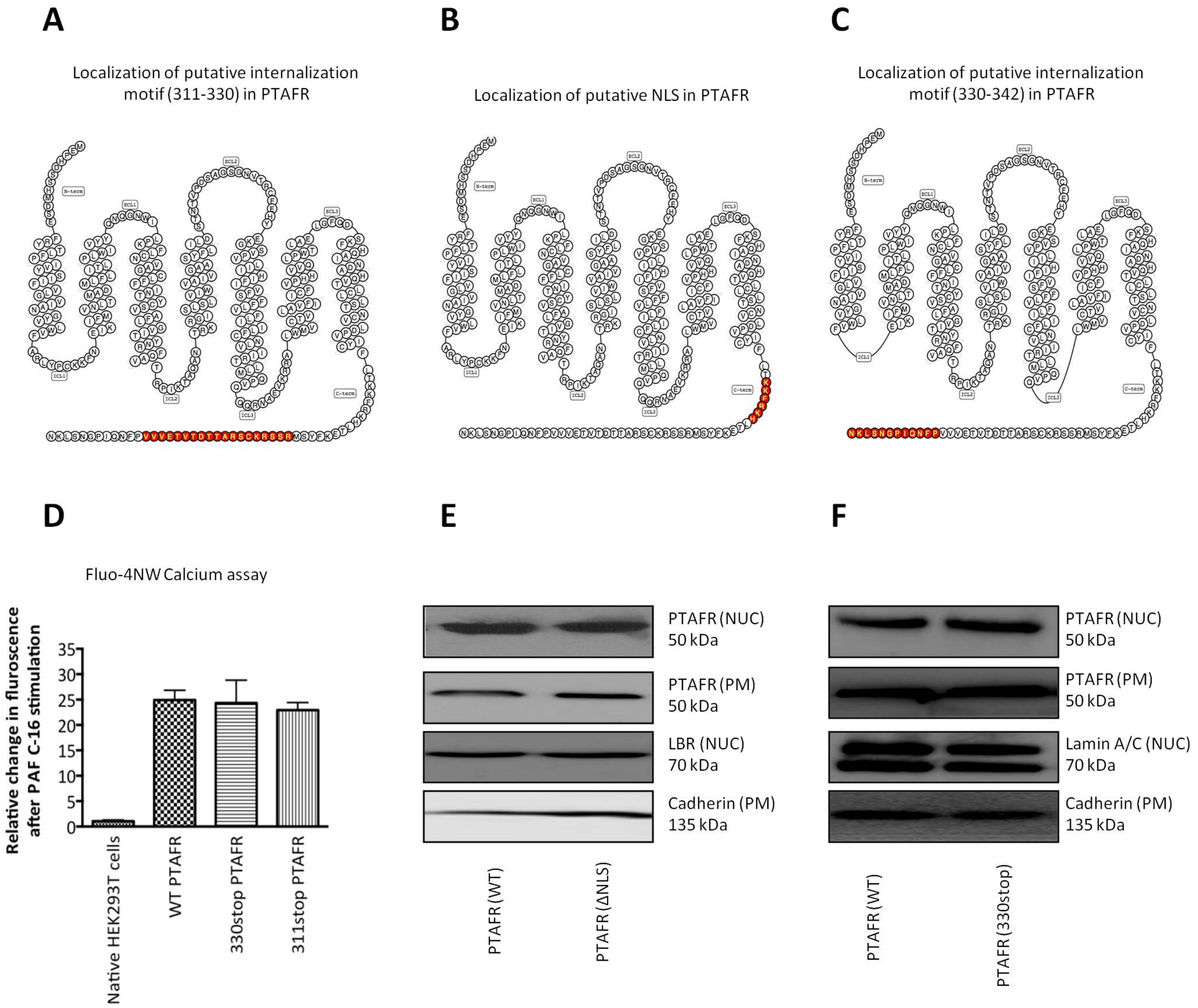

Supplemental figure-2. (**A**) (A) Schematics of Ptafr sequence to show the putative internalization motif of PTAFR between amino acid positions 311 and 330 [1]. (**B**) Localization of putative nuclear localization signal in PTAFR at positions 298 to 302 (KKFRKH) [2]. (**C**) Putative ER retention motif at the end of C-terminus of PTAFR [2]. (**D**) Calcium mobilization assay using Fluo-4NW indicator. The stimulation of HEK293T cells transfected with wild-type, 311stop and 330stop PTAFR results in intracellular rise in calcium levels, indicating that all receptors are functional. (**E**) Subcellular fractionation of CHO-K1 cells transfected with PTAFR. The mutational disruption of putative NLS from KKFRKH to NNFRKH (ΔNLS PTAFR) does not affect localization of the receptor at the cell surface or at the nucleus. (**F**) Subcellular fractionation of CHO-K1 cells transfected with PTAFR. The removal of putative ER retention motif (330stop PTAFR) also does not affect either PM or nuclear localization of PTAFR. All western blots are representative of three replicates.
