# Supplementary material for: Nuclear localization of platelet-activating factor receptor controls retinal neovascularization
Source: Cell Discov. 2016 Jul 12;2:16017–. doi: 10.1038/celldisc.2016.17 (PMC4941644; doi:10.1038/celldisc.2016.17)
Supplement: Supplementary Figure S3 [file celldisc201617-s3.pdf]

Supplemental figure-3

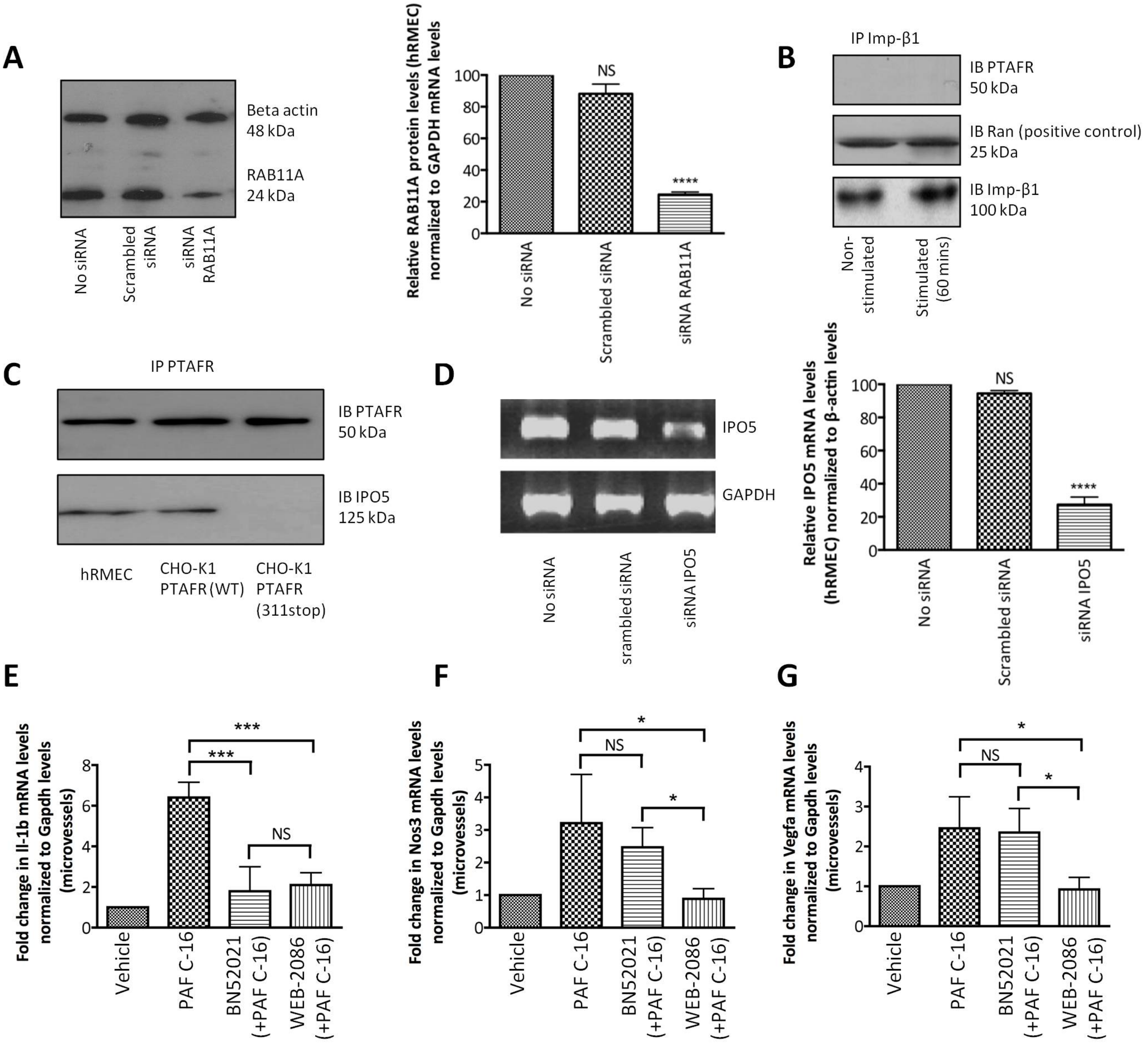

Supplemental figure-3. (**A**) RAB11A knockdown in hRMEC. There is ~65% reduction in RAB11A protein levels 72 hours following the specific siRNA treatment. The graph is average of 3 separate western blots and the representative blot is shown here. *** $p < 0.001$ (**B**) Co-immunoprecipitation of importin β1 interacting partners in hRMEC under non-stimulated and stimulated with PAF C-16 (after 60 minutes) conditions. It shows that former does not interact with PTAFR. Ran GTPase, a known interacting partner Imp β1 was used as positive control. (**C**) Co-immunoprecipitation with IPO5 in hRMEC as well as CHO-K1 cells transfected with the receptor. IPO5 co-IPs with wild type PTAFR but not with 311stop mutant receptor. (**D**) IPO5 knockdown in hRMEC. There is ~80% reduction in IPO5 mRNA levels 72 hours following the specific siRNA treatment. The graph is average of 3 separate agarose gels and the representative gels is shown. **** $p < 0.0001$ (**E**), (**F**) and (**G**) represent PAF-induced expression *Il1b*, *Nos3*, and *Vegfa* (analyzed by qRT-PCR) respectively in freshly isolated rat brain microvessels in presence or absence of PAF antagonists. The *Il1b* expression was attenuated by both PAF antagonists, while that of other two genes was only reduced by membrane permeable antagonist (WEB-2086) acting on surface as well as intracellular Ptafr. The data are represented as mean +/- s.d. (average of 3 independent qRT-PCR measurements). * $p < 0.05$, ** $p < 0.01$, and *** $p < 0.001$. NS = not significant. All blots on the figure are representative of 3 independent experiments.
