# Supplementary material for: Nuclear localization of platelet-activating factor receptor controls retinal neovascularization
Source: Cell Discov. 2016 Jul 12;2:16017–. doi: 10.1038/celldisc.2016.17 (PMC4941644; doi:10.1038/celldisc.2016.17)
Supplement: Supplementary Figure S4 [file celldisc201617-s4.pdf]

Supplemental figure-4

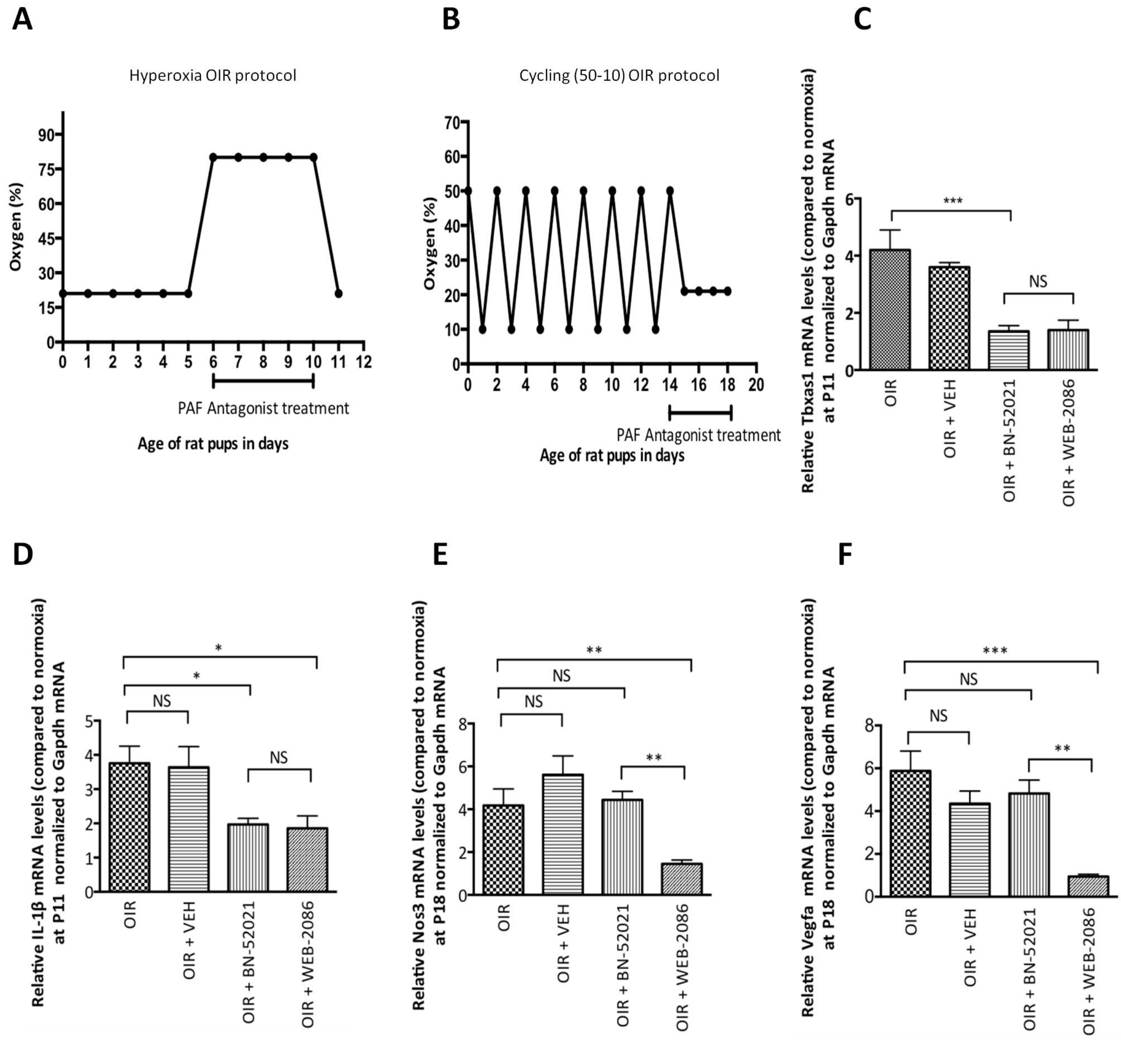

Supplemental figure-4. *In vivo* gene expression by intracellular PTAFR during two phases of OIR. (**A**) Schematic representation of hyperoxia model of OIR. (**B**) Schematic representation of cycling model of OIR. (**C**) Hyperoxia-induced upregulation of *Tbxas1 at P11. Tbxas1* mRNA levels are significantly elevated in OIR (hyperoxia) and this upregulation is prevented by administration of either PAF antagonists (from P5 to P10). (**D**) *Il1b* mRNA expression at P11 during hyperoxia model of OIR. The OIR-induced *Il1b* expression is attenuated by once daily treatment with either PAF antagonists but not that of the vehicle (DMSO). (**E**), and (**F**) Induction of pro-angiogenic genes, *Nos3* and *Vegfa* respectively, at P18 during cycling model of OIR. The OIR-induced *Nos3* and *Vegfa* levels are significantly reduced by WEB-2086 but not by BN-52021 or the vehicle treatment from P14 to P18. For sub-panels (C) to (F), the data are represented as mean +/- s.d (n= 5-8 retinas per group, NS = not significant). * $p < 0.05$, ** $p < 0.01$, and *** $p < 0.001$.
