# Supplementary material for: Nuclear localization of platelet-activating factor receptor controls retinal neovascularization
Source: Cell Discov. 2016 Jul 12;2:16017–. doi: 10.1038/celldisc.2016.17 (PMC4941644; doi:10.1038/celldisc.2016.17)
Supplement: Supplementary Table S1 [file celldisc201617-s5.pdf]

Supplemental Table-1 PTAFR Motifs and Mutagenesis

	PTAFR motif (all motifs are present in C-terminus)	Ref. for the motif	Mutagenesis	Ref. for the mutation
1.	Monopartite nuclear localization signal (NLS) ^{298}KKFRKH^{302}	[2]	^{298}NNFRKH^{302} i.e. first two lysine (K) residues were mutated to asparagine (N) residues.	[3]
2.	Putative internalization motif (last 26 amino acid residues) i.e. 317 to 342 amino acid residues	[1]	311stop PTAFR	[4]
3.	Putative ER retention motif ^{338}NLSK^{341}	[2]	330stop PTAFR	
