# Supplementary material for: Nuclear localization of platelet-activating factor receptor controls retinal neovascularization
Source: Cell Discov. 2016 Jul 12;2:16017–. doi: 10.1038/celldisc.2016.17 (PMC4941644; doi:10.1038/celldisc.2016.17)
Supplement: Supplementary Table S2 [file celldisc201617-s6.pdf]

Supplemental Table-2 Primer sequences (Human genes)

	Human Gene name	symbol	Primer sequence (5'-3')	Ref
1.	glyceraldehyde-3-phosphate dehydrogenase	*GAPDH*	Forward- 5'-CGGAGTCAACGGATTTGGTCGTAT-3' Reverse- 5'-AGCCTTCTCCATGGTGGTGAAGAC-3'	5
2.	nitric oxide synthase 3 (endothelial nitric oxide synthase)	*NOS3*	Forward- 5'-CCAGCTAGCCAAAGTCACCAT-3' Reverse- 5'-GTCTCGGAGCCATACAGGATT-3'	6
3.	RAB11A, member RAS oncogene family	*RAB11A*	Forward- 5'-TTTTGCAGAGAAGAATGGTTTGTC-3' Reverse- 5'-CCTTTGGCTTGTTCTCAGTGGT-3'	7
4.	importin 5 (karyopherin beta 3 & Ran binding protein 5)	*IPO5*	Forward- 5'-CTTTCCAGGACCCATGTGTAG-3' Reverse- 5'-CTTTCCTCTGTTGAGTGCCG-3'	
5.	vascular endothelial growth factor A	*VEGFA*	Forward- 5'-ACGAAAGCGCAAGAAATCC-3' Reverse- 5'-GGAGGCTCCAGGGCATTAG-3'	
6.	interleukin 1, beta	*IL1B*	Forward- 5'-AAACAGATGAAGTGCTCCTTCCAGG-3' Reverse- 5'-TGGAGAACACCACTTGTTGCTCCA-3'	8
