# Supplementary material for: Nuclear localization of platelet-activating factor receptor controls retinal neovascularization
Source: Cell Discov. 2016 Jul 12;2:16017–. doi: 10.1038/celldisc.2016.17 (PMC4941644; doi:10.1038/celldisc.2016.17)
Supplement: Supplementary Table S3 [file celldisc201617-s7.pdf]

Supplemental Table-3 Primer sequences (Rat genes)

	Rat Gene name	symbol	Primer sequence (5'-3')	Ref
1.	glyceraldehyde-3-phosphate dehydrogenase	*Gapdh*	Forward- 5'-CCAGGGCTGCCTTCTCTTGT-3' Reverse- 5'-CCAGCCTTCTCCATGGTGGT-3'	
2.	vascular endothelial growth factor A	*Vegfa*	Forward- 5'-CAATGATGAAGCCCTGGAGT-3' Reverse- 5'-AATGCTTTCTCCGCTCTGAA-3'	9
3.	thromboxane A synthase 1	*Tbxas1*	Forward- 5'-TCCTATAAGGGCAGGACACG-3' Reverse- 5'-GCCAAGAGAACCACGGACAG-3'	
4.	interleukin 1, beta	*Il1b*	Forward- 5'-CATCTTTGAAGAAGAGCCCG-3' Reverse- 5'-GGGATTTTGTCGTTGCTTGT-3'	9
5.	nitric oxide synthase 3	*Nos3*	Forward- 5'-ATTGGCATGAGGGACCTGTG-3' Reverse- 5'-CCGGGTGTCTAGATCCATGC-3'	

Supplemental References:

1 Le Gouill C, Parent JL, Rola-Pleszczynski M, Stankova J. Structural and functional requirements for agonist-induced internalization of the human platelet-activating factor receptor. *J Biol Chem* 1997; **272**:21289-21295.

2 Marrache AM, Gobeil F, Jr., Bernier SG *et al.* Proinflammatory gene induction by platelet-activating factor mediated via its cognate nuclear receptor. *J Immunol* 2002; **169**:6474-6481.

3 Srinivasan M, Edman CF, Schulman H. Alternative splicing introduces a nuclear localization signal that targets multifunctional CaM kinase to the nucleus. *J Cell Biol* 1994; **126**:839-852.

4 Chen Z, Dupre DJ, Le Gouill C, Rola-Pleszczynski M, Stankova J. Agonist-induced internalization of the platelet-activating factor receptor is dependent on arrestins but independent of G-protein activation. Role of the C terminus and the (D/N)PXXY motif. *J Biol Chem* 2002; **277**:7356-7362.

5 Navone SE, Marfia G, Invernici G *et al.* Isolation and expansion of human and mouse brain microvascular endothelial cells. *Nat Protoc* 2013; **8**:1680-1693.

6 Teng B, Murthy KS, Kuemmerle JF *et al.* Expression of endothelial nitric oxide synthase in human and rabbit gastrointestinal smooth muscle cells. *Am J Physiol* 1998; **275**:G342-351.

7 Gebhardt C, Breitenbach U, Richter KH *et al.* c-Fos-dependent induction of the small ras-related GTPase Rab11a in skin carcinogenesis. *Am J Pathol* 2005; **167**:243-253.

8 Keita M, Bessette P, Pelmus M, Ainmelk Y, Aris A. Expression of interleukin-1 (IL-1) ligands system in the most common endometriosis-associated ovarian cancer subtypes. *Journal of ovarian research* 2010; **3**:3.

9 Rivera JC, Sitaras N, Noueihed B *et al.* Microglia and interleukin-1beta in ischemic retinopathy elicit microvascular degeneration through neuronal semaphorin-3A. *Arterioscler Thromb Vasc Biol* 2013; **33**:1881-1891.
